# Blood-Based Indicators of Insulin Resistance and Metabolic Syndrome in Bottlenose Dolphins (*Tursiops truncatus*)

**DOI:** 10.3389/fendo.2013.00136

**Published:** 2013-10-09

**Authors:** Stephanie Venn-Watson, Cynthia Rowe Smith, Sacha Stevenson, Celeste Parry, Risa Daniels, Eric Jensen, Veronica Cendejas, Brian Balmer, Michael Janech, Benjamin A. Neely, Randall Wells

**Affiliations:** ^1^Translational Medicine and Research Program, National Marine Mammal Foundation, San Diego, CA, USA; ^2^Navy Marine Mammal Program, Space and Naval Warfare Systems Center Pacific, San Diego, CA, USA; ^3^Sarasota Dolphin Research Program, Chicago Zoological Society c/o Mote Marine Laboratory, Sarastota, FL, USA; ^4^Division of Nephrology, Department of Medicine, Medical University of South Carolina, Charleston, SC, USA

**Keywords:** adiponectin, bottlenose dolphin, diabetes, insulin resistance, iron, metabolic syndrome

## Abstract

Similar to people with metabolic syndrome, bottlenose dolphins (*Tursiops truncatus*) can have a sustained postprandial hyperglycemia and hyperinsulinemia, dyslipidemia, and fatty liver disease. A panel of potential postprandial blood-based indicators of insulin resistance and metabolic syndrome were compared among 34 managed collection dolphins in San Diego Bay, CA, USA (Group A) and 16 wild, free-ranging dolphins in Sarasota Bay, FL, USA (Group B). Compared to Group B, Group A had higher insulin (2.1 ± 2.5 and 13 ± 13 μIU/ml), glucose (87 ± 19 and 108 ± 12 mg/dl), and triglycerides (75 ± 28 and 128 ± 45 mg/dl) as well as higher cholesterol (total, high-density lipoprotein cholesterol, and very low density lipoprotein cholesterol), iron, transferrin saturation, gamma-glutamyl transpeptidase (GGT), alanine transaminase, and uric acid. Group A had higher percent unmodified adiponectin. While Group A dolphins were older, the same blood-based differences remained when controlling for age. There were no differences in body mass index (BMI) between the groups, and comparisons between Group B and Group A dolphins have consistently demonstrated lower stress hormones levels in Group A. Group A dolphins with high insulin (greater than 14 μIU/ml) had higher glucose, iron, GGT, and BMI compared to Group A dolphins with lower insulin. These findings support that some dolphin groups may be more susceptible to insulin resistance compared to others, and primary risk factors are not likely age, BMI, or stress. Lower high-molecular weight adiponectin has been identified as an independent risk factor for type 2 diabetes in humans and may be a target for preventing insulin resistance in dolphins. Future investigations with these two dolphin populations, including dietary and feeding differences, may provide valuable insight for preventing and treating insulin resistance in humans.

## Introduction

An estimated 347 million people have diabetes globally with an expected increase to 450 million people in less than 20 years (1, 2). While type 2 diabetes (T2D) represents 90% of these cases, type 1 diabetes (T1D) is also on the rise, and the search for a cure for both is urgent (3). Investigating naturally occurring or incidental metabolic diseases in non-traditional animal populations may provide valuable insight related to the evolution of, risks for, and protection against both T1D and T2D in humans (4, 5).

**Figure 1:**
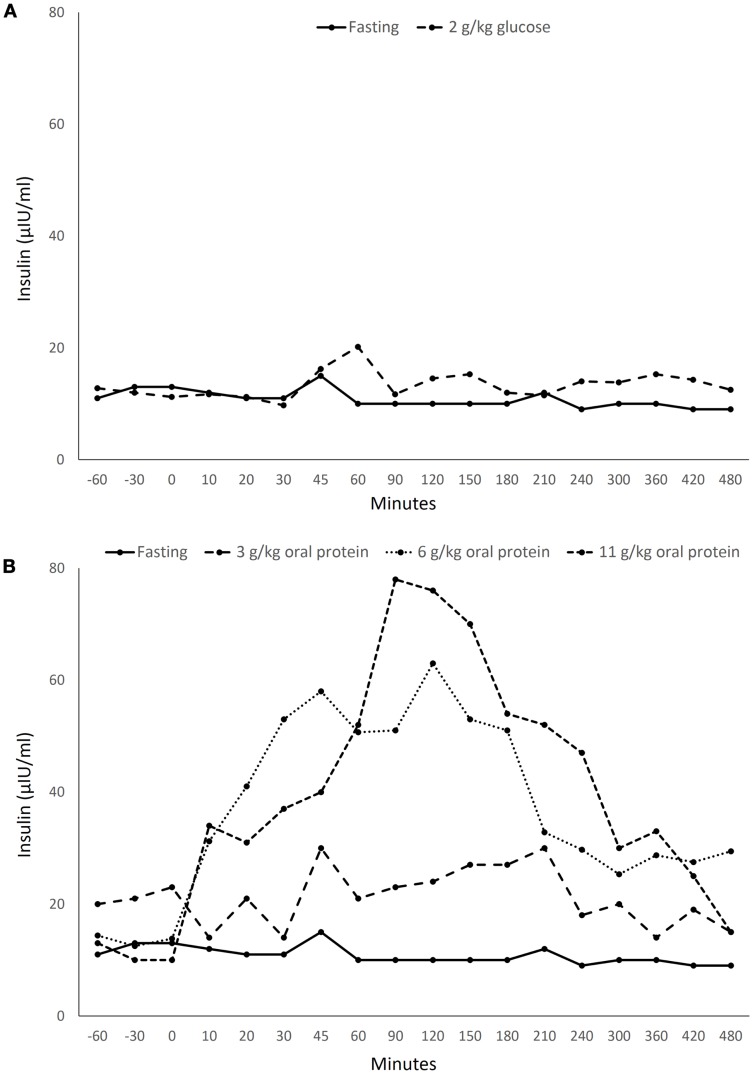
**Insulin levels in a healthy bottlenose dolphin (*Tursiops truncatus*) following ingestion of (A) 2 g/kg oral glucose and (B) 3–11 g/kg protein from Spanish mackerel**. Data acquired from Patton (8) with permission from the U.S. Navy.

Bottlenose dolphins (*Tursiops truncatus*), similar to humans with diabetes, can exhibit a sustained postprandial hyperglycemia (6, 7). After oral glucose administration, dolphins have a sustained hyperglycemia paired with a negligible insulin response, similar to that found in T1D (insulin depletion) (8; Figure 1A). When dolphins ingest a large fish meal, however, they have a sustained hyperglycemic and hyperinsulinemic response, matching that of a person with early T2D (insulin resistance) (8; Figure 1B). Dolphins are also susceptible to diseases and conditions associated with insulin resistance in humans, including fatty liver disease, urate nephrolithiasis, hemochromatosis, and chronic elevations in triglycerides, cholesterol, and indicators of inflammation (9–12). Thus, dolphins may provide new insights for metabolic syndrome, T1D, and T2D.

Some groups of people are more susceptible to diabetes compared to others (13). In addition to diet and body mass index (BMI), proposed or known risk factors include genetics, work schedules, race and ethnicity, and geographic locations (14–17). Similar to humans, it has been proposed that some dolphin groups may be at a higher risk of developing metabolic disease compared to others (18). Hypothesized reasons for metabolic perturbations in dolphins include older age, overnight fasting, larger meal sizes with higher purine loads, work schedules that may vary from their natural circadian rhythm, and genetics (10). If dolphin populations can be identified that have a relatively high and low risk of insulin resistance and metabolic syndrome, comparing these populations may help identify causes, and more importantly, means of preventing insulin resistance and metabolic syndrome.

Two groups of dolphins that have been well studied and compared over the past 40 years are the wild, free-ranging dolphins in Sarasota Bay, FL, USA, and the U.S. Navy’s managed collection dolphins in San Diego Bay, CA, USA (19, 20). Potential blood-based indicators of insulin resistance and metabolic disease in dolphins were compared among postprandial samples collected from these two dolphin groups.

## Materials and Methods

### Managed and free-ranging group comparisons

The two populations in this study were managed collection Atlantic bottlenose dolphins living in San Diego Bay, CA, USA, cared for by the Navy Marine Mammal Program (MMP) (Group A, *n* = 34) and free-ranging Atlantic bottlenose dolphins living in Sarasota Bay, FL, USA (Group B, *n* = 16). The Navy has housed and cared for dolphins for over 50 years and has over 1,000 peer-reviewed scientific publications with this population. MMP dolphins live in netted enclosures within San Diego Bay, many of which have daily open ocean activity sessions. They are fed high-quality, frozen-thawed whole fish diets consisting of primarily capelin and herring, as well as squid and mackerel. Diets for individual dolphins are based upon kilocalories by body weight. MMP dolphins are typically fed their daily intake over three to eight meals between 0800 and 1500. Before 1990, most MMP dolphins originated from the Gulf of Mexico, especially Mississippi Sound. Since the early 1990s, MMP dolphins have been born at the MMP facility in San Diego Bay. Involvement of MMP dolphins for this study was approved by the MMP’s Institutional Animal Care and Use Committee (IACUC) and the Navy Bureau of Medicine (Protocol # 101-2012). The MMP is accredited by the Association for Assessment and Accreditation of Laboratory Animal Care International.

Sarasota Bay dolphins have been studied since 1970; the long-term resident population in 2013 spans up to five generations and includes males up to 50 years old and females up to 63 years old (20, 21). Descriptions of feeding and activity patterns of Sarasota Bay dolphins are provided in Wells et al. (22). Wild dolphin sampling was approved by the Mote Marine Laboratory IACUC. Blood sampling from wild dolphins was approved under National Marine Fisheries Service Scientific Research Permit No. 522-1785 issued to RSW.

The two study populations are both from the Gulf of Mexico but from different stocks. They are both coastal feeding dolphins and are taxonomically the same species.

### Feeding and diet

Group A was fed one-third of their daily diet in the morning after their routine overnight fast. Routine diets are based upon kilocalories per kilogram of body weight and pre-established caloric needs that vary by age, sex, and activity of each dolphin. Fish fed were restaurant quality and frozen-thawed and included primarily capelin (*Mallotus villosus*) and herring (*Clupea harengus*) with or without mackerel (*Scomber japonicus*) and squid (*Loligo opalescens*). Mean ± SD of calories and macronutrients in the morning meal were 3,253 ± 732 kcal, 453 ± 132 g protein, 184 ± 43 g fat, and 1 ± 1 g of carbohydrates.

While the timing of the most recent meal prior to each Group B dolphin’s capture-release was unknown, sonography was used to assess the presence or absence of stomach contents; Group B dolphins in the study had contents in their stomachs, supporting they were in a postprandial state. A summary of diets and feeding behaviors in free-ranging dolphins living in Sarasota Bay is provided in Wells et al. (22).

### Sample collection

#### Group A

Following the morning meal, 2 h postprandial blood samples were drawn (typically near 10:00 a.m.). These dolphins were trained to present their flukes for blood collection, or blood was collected from the peduncle while the animal rested on a foam mat out of water during a routine physical exam.

#### Group B

Dolphins were captured in shallow water with a seine net at similar times of the day (20). Following sample collection, animals were released on site. Blood samples were collected from the fluke while the animal was restrained in the water or aboard a specially designed veterinary examination vessel.

#### Sampling protocol for both study groups

Blood samples were collected using 19–21 gage, 1.5″ Vacutainer^®^ needles (Becton Dickinson VACUTAINER Systems, Rutherford, NJ, USA 07070) or a 21 gage, 0.75″ butterfly needle attached to a Vacutainer^®^ Holder.

Blood collected into EDTA was used for complete blood cell count, 60 min erythrocyte sedimentation rate (ESR), and glucagon levels. EDTA tubes for glucagon were chilled and 0.04 cc of aprotinin per milliliter of whole blood collected was added immediately. Blood was centrifuged at 4°C within 30 min and plasma was transferred to −80°C storage within 1 h. Centrifugation was performed at 3,000 rpm at 4°C for 10 min. Blood collected into Lithium Heparin was utilized for the lipid panel. Samples were chilled for 30 min and centrifuged within 2 h. Samples were sent on cold packs or dry ice via overnight courier to the reference laboratories. Blood collected into serum separator tubes was utilized for serum chemistry, insulin, free fatty acids, haptoglobin, and adiponectin. Blood for serum chemistry was left at room temperature and centrifuged within 1 h. Blood for insulin, free fatty acids, haptoglobin, and adiponectin was chilled for 30 min, centrifuged within 2 h, and transferred to 80°C storage.

### Diagnostic tests

Total white blood cell (WBC) count was calculated using automated complete blood count analyses on the Beckman Coulter LH755 (Beckman Coulter, Brea, CA, USA). Triglycerides and high-density lipoprotein cholesterol (HDL-C) were directly measured using the Roche Cobas 8000 system (Roche Molecular Systems, Pleasanton, CA, USA). Very low density lipoprotein cholesterol (VLDL-C) was calculated using the following equation: [triglycerides/5]. Low density lipoprotein cholesterol (LDL-C) cholesterol was calculated as [cholesterol-(VLDL + HDL)]. Fisherbrand Dispette 2^®^, correlating with the Westergren method, was used to determine 60 min ESR in-house). Automated complete blood count analyses and cholesterol measurements were performed by the Naval Medical Center San Diego.

Serum chemistry analytes included glucose, creatinine, uric acid, carbon dioxide (CO_2_), anion gap, globulins, alkaline phosphatase (ALP), lactate dehydrogenase (LDH), aspartate aminotransferase (AST), alanine aminotransferase (ALT), gamma-glutamyl transpeptidase (GGT), and iron. The above variables were measured photometrically on the Roche Diagnostics Modular Analytics P Module clinical chemistry analyzer (Roche Diagnostics, Indianapolis, IN, USA) at the Animal Health Diagnostic Center at Cornell University. Adiponectin was measured using parallel reaction monitoring (PRM) as described previously (23).

Additionally, free and total insulin, free fatty acids, glucagon, and haptoglobin were analyzed. Both free and total insulin were measured by ultrafiltration/quantitative chemiluminescent immunoassay on the Siemens Advia Centaur^®^ Immunoassay system (Bayer Diagnostics/Siemens Medical Solutions Diagnostics, Tarrytown, NY, USA). Free fatty acids were measured spectrophotometrically on the Roche Cobas c501 (Roche Diagnostics, Indianapolis, IN, USA). Glucagon was measured by double antibody radioimmunoassay using the DPC Glucagon Double Anti body Kit (Siemens, Tarrytown, NY, USA). Insulin, free fatty acids, and glucagon were analyzed at ARUP laboratory. Serum haptoglobin was measured spectrophotometrically at Kansas State Veterinary Diagnostic Lab (24).

### Statistical analyses

Analyses were conducted using SAS Version 9.2 (SAS Incorporated, Cary, NC, USA). Quantile values for Group A dolphins were determined for each blood-based variable. Elevated insulin levels were defined as values greater than the 75th quantile of the study’s Group A dolphins. Age, sex, BMI [weight (km)/length^2^ (m)] and blood values were compared among the following groups: (1) Group A and Group B, and (2) Group A dolphins with and without elevated insulin. Sex distribution was compared using a Mantel–Haenszel Chi-square test; when low numbers were involved, a Fisher’s exact test was used. Age and blood variable values were compared using a Wilcoxon rank-sum test. Since Group A dolphins were significantly older than Group B dolphins, a covariate analysis using a general linear model was used, controlling for age, to compare the two groups. In all analyses, significance was defined as a *P* value less than 0.05.

## Results

### Comparisons of values between populations

There was no significant difference in sex distribution between the study groups (percent female, Group A = 44%, Group B = 31%; *P* = 0.39). Group A dolphins were older than Group B dolphins (mean ages = 24.5 and 14 years, respectively; *P* = 0.003). There were no differences in BMI when comparing Group A and Group B (29 ± 3 and 28 ± 6, respectively; *P* = 0.19). Compared to Group B, Group A dolphins were more likely to have higher glucose, free and total insulin, triglycerides, total cholesterol, HDL-C, VLDL-C, iron, transferrin saturation, GGT, ALT, uric acid, and percent unmodified adiponectin. Group B dolphins were more likely to have higher free fatty acids, WBC counts, and globulins compared to Group A dolphins (Table 1).

**Table 1:** **Comparisons of potential metabolic, blood-based biomarkers between a managed collection and a wild, free-ranging bottlenose dolphin (*Tursiops truncatus*) population**.

Blood variable	Mean value ± SD	
	Group A Managed collection (*n* = 34)	Group B Free-ranging (*n* = 16)
Glucose (mg/dl)	108 ± 12*	87 ± 19
Insulin, free (μIU/ml)	10 ± 10*	1.9 ± 2.0
Insulin, total (μIU/ml)	13 ± 13*	2.1 ± 2.5
Glucagon (pg/ml)	170 ± 64	152 ± 93
Free fatty acids (mmol/l)	0.4 ± 0.2	1.4 ± 0.5*
**Lipids**
Triglycerides (mg/dl)	128 ± 45*	75 ± 28
Cholesterol – total (mg/dl)	217 ± 51*	155 ± 29
HDL-C (mg/dl)	175 ± 33*	122 ± 17
LDL-C (mg/dl)	23 ± 25	19 ± 18
VLDL-C (mg/dl)	26 ± 9*	15 ± 5
**Inflammation**
WBC (cells/μl × 10^−3^)	7.5 ± 1.6	10.0 ± 1.9*
Globulins (g/dl)	1.8 ± 0.4	2.7 ± 0.4*
ESR (mm/h)	15 ± 12	13 ± 14
Haptoglobin (mg/dl)	581 ± 238	616 ± 268
Total adiponectin	572 ± 3	615 ± 6
Unmodified adiponectin (%)	20 ± 8*	11 ± 5
**Iron metabolism and liver**
Iron (μg/dl)	178 ± 62*	120 ± 28
Transferrin saturation (%)	59 ± 20*	41 ± 8
GGT (U/l)	30 ± 15*	19 ± 4
AST (U/l)	286 ± 88	246 ± 39
ALT (U/l)	51 ± 20*	36 ± 15
LDH (U/l)	475 ± 86	479 ± 79
**Renal function and acid-base balance**
Creatinine (mg/dl)	1.1 ± 0.2	1.3 ± 0.4
CO_2_ (mEq/l)	29 ± 4	28 ± 3
Uric acid (mg/dl)	0.7 ± 0.3*	0.4 ± 0.3

*Significantly higher (*P* < 0.05).

Higher WBC counts in Group B dolphins were due to higher eosinophil counts compared to managed collection dolphins (3,181 ± 1,561 and 1,050 ± 539 cells/μl, respectively; *P* < 0.0001); neutrophils, monocytes, and lymphocytes were not higher in Group B compared to Group A dolphins. Group A dolphins were more likely to have high transferrin saturation (greater than 60%) compared to Group B dolphins (14/34, 41.2% and 0/16, respectively; *P* = 0.0003). Group A dolphins were also more likely to have elevated glucose (greater than 122 mg/dl) (7/34, 20.6% and 0/16, respectively; *P* = 0.02).

When controlling for age, Group A dolphins continued to have higher glucose, free and total insulin, triglycerides, total cholesterol, HDL-C, VLDL-C, iron, transferrin saturation, GGT, ALT, uric acid, and percent unmodified adiponectin. Group B dolphins also still had higher free fatty acids, WBC counts, and globulins compared to Group A dolphins.

### Comparisons of Group A dolphins with elevated or non-elevated total insulin

Quantile values of the panel variables from Group A dolphins are provided (Table 2). Eight (23.5%) of 34 dolphins had elevated insulin (greater than 14 μIU/ml). There were no significant differences in sex distribution or age between dolphins with and without elevated insulin (percent female = 2/8, 25% and 13/26, 50%, *P* = 0.21; mean age = 30.5 and 22.9 years, *P* = 0.10). Dolphins with elevated insulin had higher BMI compared to those without elevated insulin (31 ± 3 and 28 ± 3, *P* = 0.01). Dolphins with elevated insulin had higher glucose, iron, and GGT compared to dolphins without elevated insulin (Table 3).

**Table 2:** **About 2 h postprandial values of potential metabolic disease biomarkers for selected quantiles among 34 managed collection bottlenose dolphins (*Tursiops truncatus*)**.

Blood variable	Quantile value				
	10th	25th	50th	75th	90th
Glucose (mg/dl)	94	99	107	114	124
Insulin, free (μIU/ml)	3	5	8	11	23
Insulin, total (μIU/ml)	4	4	9	14	27
Glucagon (pg/ml)	97	116	175	198	262
Free fatty acids (mmol/l)	0.29	0.32	0.40	0.48	0.55
**Lipids**
Triglycerides (mg/dl)	80	97	121	151	194
Cholesterol – total (mg/dl)	157	171	207	252	283
HDL-C (mg/dl)	134	147	174	205	218
LDL-C (mg/dl)	3	4	15	37	51
VLDL-C (mg/dl)	16	19	24	30	39
**Inflammation**
WBC (cells/μl × 10^−3^)	5.9	6.3	7.4	8.4	9.1
Globulins (g/dl)	1.4	1.6	1.8	2.0	2.5
ESR (mm/h)	2	4	6	12	20
Haptoglobin (mg/dl)	3.4	6.6	12	22	32
Total adiponectin	286	393	525	688	948
Unmodified adiponectin %	11	14	19	24	32
**Iron metabolism and liver**
Iron (μg/dl)	106	135	174	215	264
Transferrin saturation (%)	35	44	59	72	92
GGT (U/l)	19	22	26	32	42
**Renal function and acid-base balance**
Creatinine (mg/dl)	0.8	1.0	1.1	1.3	1.4
CO_2_ (mEq/l)	26	27	28	30	38
Anion gap	12	14	15	16	17
Uric acid (mg/dl)	0.4	0.5	0.6	0.8	1.0

**Table 3:** **Comparisons of selected blood variable values among dolphins with and without elevated 2 h postprandial total insulin levels ( >14 μIU/ml)**.

Blood variable	Mean value ± SD	
	Elevated total insulin (*n* = 8)	Non-elevated total insulin (*n* = 26)
Glucose (mg/dl)	116 ± 12*	106 ± 12
Insulin, free (μIU/ml)	24 ± 12*	6.1 ± 2.4
Insulin, total (μIU/ml)	31 ± 16*	7.7 ± 3.6
Glucagon (pg/ml)	147 ± 46	178 ± 67
Free fatty acids (mmol/l)	0.4 ± 0.1	0.5 ± 0.2
**Lipids**
Triglycerides (mg/dl)	148 ± 59	123 ± 39
Cholesterol – total (mg/dl)	211 ± 24	219 ± 58
HDL-C (mg/dl)	185 ± 24	172 ± 35
LDL-C (mg/dl)	11 ± 6	25 ± 26
VLDL-C (mg/dl)	30 ± 12	25 ± 8
**Inflammation**
WBC (cells/μl × 10^−3^)	7.6 ± 2.3	7.4 ± 1.3
Globulins (g/dl)	1.7 ± 0.5	1.9 ± 0.4
ESR (mm/h)	11 ± 9	9 ± 9
Haptoglobin (mg/dl)	12 ± 12	16 ± 12
Total adiponectin	594 ± 237	576 ± 244
Unmodified adiponectin (%)	16 ± 7	22 ± 8
**Iron metabolism and liver**
Iron (μg/dl)	213 ± 32*	167 ± 66
Transferrin saturation (%)	68 ± 15	56 ± 21
GGT (U/l)	34 ± 9*	29 ± 16
AST (U/l)	282 ± 68	287 ± 94
ALT (U/l)	53 ± 22	51 ± 19
LDH (U/l)	498 ± 78	468 ± 89
**Renal function and acid-base balance**
Creatinine (mg/dl)	1.2 ± 0.2	1.1 ± 0.2
CO_2_ (mEq/l)	29 ± 3.8	28 ± 4.0
Uric acid (mg/dl)	0.6 ± 0.2	0.7 ± 0.3

*Significantly higher (*P* < 0.05).

## Discussion

In the current study, common blood-based indicators of insulin resistance and metabolic syndrome in humans were compared between two groups of dolphins. Postprandial glucose, insulin, cholesterol, and triglycerides were higher in the managed dolphin group compared to the free-ranging dolphin group. Among managed dolphins, those with elevated insulin levels (greater than 14 μIU/ml) had higher glucose, GGT, iron, and BMI compared to managed dolphins without elevated insulin. Previous studies have demonstrated that approximately 39 and 67% of this study’s managed population have subclinical fatty liver disease and hemochromatosis at death (10). Metabolic syndrome in humans has a variety of definitions, but most include high postprandial glucose, hyperinsulinemia, hypertriglyceridemia, and non-alcoholic fatty liver disease (NAFLD) (25–27). As such, this study supports that some groups of dolphins may be more susceptible to insulin resistance, metabolic syndrome, and their complications compared to others.

Free-ranging dolphins from Sarasota Bay, FL, USA, and managed dolphins at the Navy MMP in San Diego, CA, USA included in this study have been well studied for over 40 years (19, 20). Many Sarasota Bay dolphins demonstrate strong fidelity to their location and group, and health is tracked throughout each dolphin’s lifetime using routine population surveys and capture-release health assessments. The MMP cares for a population of approximately 80 dolphins that receive top-level care from a team of marine mammal veterinarians, are trained to provide routine, voluntary blood samples, and are part of a vigilant preventive health program.

Assessed differences between these two groups of dolphins have included age, BMI, and stress hormones. Annual survival rates and mean age at death of Sarasota Bay and MMP dolphins are 0.97 and 0.99; and 19.9 and 36 years old, respectively (22, 28, 29). While MMP dolphins in this study were older than Sarasota Bay dolphins, differences in blood values between these two groups remained after controlling for age. With regard to BMI, there was no difference between the two study groups. Previous comparisons have demonstrated that Sarasota Bay dolphins have significantly higher levels of stress hormones cortisol and aldosterone compared to MMP dolphins (30). This comparison was again confirmed with current data, in which the mean cortisol level in Sarasota Bay dolphins during the study was 2.99 μg/dl; the mean level recently measured among 29 MMP dolphins was less than 0.43 μg/dl (unpublished data, personal communication with Houser). Thus, increased free fatty acid levels identified in Sarasota Bay dolphins were possibly due to relatively higher stress; both cortisol and free fatty acid levels can increase during stress (31). This is not surprising, given that free-ranging dolphins are captured and released, while MMP dolphins are trained to voluntarily and routinely present their flukes for blood sampling. Thus, old age, high BMI, and stress are not supported as leading causes of insulin resistance and metabolic syndrome in dolphins.

Diet is an important risk factor for insulin resistance and metabolic syndrome in humans (32). Sarasota Bay dolphins eat a wide variety of live fish and squid, while MMP dolphins are fed high-quality frozen-thawed fish, primarily capelin and herring (22). Nutritional content comparisons of fish commonly ingested by Sarasota Bay versus MMP dolphins (pigfish, pinfish, and mullet versus capelin and herring) suggested that Sarasota Bay fish may have higher calcium and vitamin D; lower potassium, sodium, zinc, linoleic acid, and docosahexaenoic acid; and differences in polyunsaturated fatty acids compared to fish eaten by managed dolphins (33). Interestingly, fish diets have been found to be both beneficial and detrimental for people with type 2 diabetes (34, 35). Further studies in dolphins and their diets may provide insight into which fish nutrients are protective against or risk factors for T2D in people.

In humans, exercise increases glycemic control and protects against the development of T2D (36). Sarasota Bay dolphins follow a consistent circadian pattern of increased activity during the day. They remain active throughout the day and rest only 2% of that time (22). They also routinely dive during the day and night and travel during the day and night at rates around 1.4 m/s (37). MMP dolphins are also actively swimming during the day and night in their large Bay-based enclosures, including daily open ocean sessions in San Diego Bay for many MMP dolphins. While similar BMI between the two groups indicates that lack of activity and obesity are not the drivers for their metabolic differences, further studies are needed to better compare energy expenditure between these two groups.

Several compelling parallels with human diabetes, insulin resistance, and metabolic syndrome were identified in this study with dolphins. Components of insulin resistance-associated dyslipidemia in humans include over production of VLDL-C and increased triglyceride levels (38). These changes, paired with moderate liver-specific insulin resistance, can lead to hepatic steatosis (39). In the current study, MMP dolphins had higher triglycerides, total cholesterol, HDL-C, and VLDL-C. Fatty liver disease in dolphins has been previously associated with chronic hyperglycemia, and findings from this study further support that dolphins and humans with metabolic syndrome may have parallel susceptibilities to dyslipidemias (10).

In the current study, paired indicators of insulin resistance with increased liver enzymes may increase the relevance of the dolphin as a model for metabolic syndrome and insulin resistance for humans. GGT and ALT were higher when comparing managed collection dolphins with the free-ranging dolphins; additionally, within the managed collection study group, GGT was higher among those with elevated insulin compared to those without elevated insulin. Increased ALT and GGT are indicators of NAFLD and its progression to non-alcoholic steatohepatitis (NASH) (40). The association between GGT and fatty liver disease in dolphins could be confirmed if liver biopsies were available for histologic examination.

Adiponectin is a protein primarily secreted by adipocytes in mammals, and it has a role in increasing insulin sensitivity and decreasing inflammation (41, 42). In the current study, managed dolphins had higher percent unmodified adiponectin compared to free-ranging dolphins. It is known that the degree of modification of adiponectin is what results in adiponectin oligomerization into mid- and high-molecular weight multimers (43, 44). While the components of modified adiponectin could not be confirmed, results from this study suggest that mid to high-molecular weight adiponectin levels were lower in managed dolphins. In humans, lower high-molecular weight adiponectin is related to insulin resistance and a higher risk for T2D (45, 46). Furthermore, lower adiponectin concentration is an independent risk factor for the development of and progression to T2D (47, 48). While further work is needed to confirm that dolphins from the managed collection in this study had lower levels of high-molecular weight adiponectin, this early finding provides a targeted area for future research.

Insulin resistance in humans has been associated with iron overload (49, 50). In the current study, 41% of managed collection dolphins tested had a transferrin saturation greater than 60% while none of the free-ranging dolphins had similarly high levels. Previous studies have demonstrated that this study’s and other managed collection dolphins have higher iron and transferrin saturation compared to free-ranging dolphin populations living in the Indian River Lagoon, FL, USA and Charleston, SC, USA (18). Hemochromatosis, or iron overload, has been well documented in MMP dolphins (9, 10). Dolphins with hemochromatosis have higher 2 h postprandial insulin levels compared to healthy controls, and approximately 67% of MMP dolphins appear to have hemochromatosis at time of death (7, 10). While none of the managed collection dolphins in the current study had clinically high iron levels (greater than 300 μg/dl), this study continues to support that elevated insulin is associated with increasing iron in dolphins. The underlying mechanism of this association in humans has not yet been fully elucidated, and better understanding of insulin and iron metabolism could benefit both dolphins and humans.

In summary, two groups of dolphins were identified that appear to have higher and lower risks of developing insulin resistance and metabolic syndrome. This study and the supporting literature did not support that age, body mass, or stress were primary drivers of insulin resistance or metabolic syndrome in dolphins. Understanding risk and protective factors for metabolic syndrome in these two dolphin populations may lead to immediate and translational means of preventing and treating insulin resistance and T2D in humans.

## Conflict of Interest Statement

The authors declare that the research was conducted in the absence of any commercial or financial relationships that could be construed as a potential conflict of interest.
